# Incremental value of placental miR-7641 and ferroptosis-related phenotypes in risk stratification of severe preeclampsia

**DOI:** 10.3389/fphys.2026.1834708

**Published:** 2026-05-13

**Authors:** Huihui Hu, Rumin Wang, Xiaodi Wang, Min Hu

**Affiliations:** Department of Obstetrics and Gynecology, The First People's Hospital of Yongkang, Jinhua, China

**Keywords:** ferroptosis, incremental value, miR-7641, net reclassification improvement, severe preeclampsia

## Abstract

**Objective:**

To evaluate the incremental value of placental miR-7641 and ferroptosis-related molecular phenotypes beyond mean arterial pressure (MAP) for risk stratification of severe preeclampsia (SPE).

**Methods:**

This retrospective study enrolled 64 women with SPE and 70 normotensive controls who underwent cesarean delivery between January 2023 and October 2024. Placental miR-7641 was quantified by qRT-PCR, and MDA, COX2, and GPX4 were measured by ELISA/TBA assay. Independent predictors were identified through multivariable logistic regression, and nested models were constructed (Model 1: MAP alone; Model 2: MAP plus independently significant molecular markers). Incremental discrimination was assessed using the net reclassification improvement (NRI), integrated discrimination improvement (IDI), and DeLong test. The SPE group was further stratified by gestational age at delivery into early-onset PE (EOPE; <34 weeks, n = 21) and late-onset PE (LOPE; ≥34 weeks, n = 43) for exploratory subgroup analysis.

**Results:**

Compared with controls, SPE placentas exhibited significantly elevated miR-7641, MDA, and COX2 levels alongside significantly reduced GPX4 (all P < 0.001), with more pronounced alterations in the EOPE subgroup (all Cohen’s d > 0.8). In multivariable analysis, MAP (OR = 1.12), miR-7641 (OR = 2.24), and MDA (OR = 1.45) were independently associated with SPE. Model 2 improved the AUC from 0.843 to 0.906 compared with Model 1 (P = 0.008), with an NRI of 0.573 (P < 0.001) and an IDI of 0.112 (P < 0.001). Sensitivity analyses demonstrated that the associations attenuated but persisted after adjustment for gestational age, supporting the robustness of the findings.

**Conclusions:**

Placental miR-7641 and MDA provide incremental risk stratification information beyond MAP, with ferroptosis-related molecular phenotypes being more prominent in EOPE. These findings constitute proof of concept; clinical translation awaits external validation.

## Introduction

Severe preeclampsia (SPE) remains a leading cause of maternal and perinatal morbidity and mortality worldwide, complicating approximately 2%–8% of pregnancies (Bonovas and Piovani, 2023; Dimitriadis et al., 2023). SPE is characterized by marked clinical heterogeneity: early-onset SPE (EOPE; delivery <34 weeks of gestation) is typically associated with prominent placental hypoperfusion and dysfunction, conferring substantially elevated risks of adverse perinatal outcomes, whereas late-onset SPE (LOPE; delivery ≥34 weeks) is more closely linked to maternal metabolic derangements and pre-existing cardiovascular risk factors, with relatively milder placental pathology (Staff et al., 2013; Staff et al., 2022). Current risk assessment strategies rely predominantly on blood pressure parameters, proteinuria, fetal growth monitoring, and the sFlt-1/PlGF ratio (Verlohren et al., 2022; Stepan et al., 2023), which provide valuable yet incomplete information for diagnosis and short-term risk prediction. A critical gap remains: these approaches are insufficient to precisely differentiate the heterogeneity between EOPE and LOPE, to quantify placental pathological burden at the molecular level, or to identify the subpopulations at highest risk for adverse maternal–fetal outcomes. Whether incorporating placental molecular information into risk assessment can provide incremental stratification value beyond conventional clinical parameters such as MAP has not been systematically evaluated.

Ferroptosis is an iron-dependent form of regulated cell death driven by lipid peroxidation and the collapse of antioxidant defense systems, mechanistically distinct from apoptosis and necroptosis (Dixon et al., 2012; Jiang et al., 2021). A growing body of evidence implicates ferroptosis in placental ischemia–reperfusion injury and oxidative stress in preeclampsia (Beharier et al., 2021; Lekva et al., 2024; Park et al., 2025). Key phenotypic hallmarks of ferroptosis include dysfunction of glutathione peroxidase 4 (GPX4)—the principal enzyme that reduces toxic lipid hydroperoxides to non-toxic alcohols—accumulation of malondialdehyde (MDA), a stable end-product indicator of lipid peroxidation, and altered expression of cyclooxygenase-2 (COX2), an inducible enzyme in oxidative-stress–driven prostaglandin synthesis (Tang et al., 2021). MicroRNAs (miRNAs)—short (~22-nucleotide) non-coding RNAs that suppress target gene expression by binding to the 3′-untranslated regions of messenger RNAs—play critical roles in the post-transcriptional regulation of placental redox homeostasis, and multiple miRNAs have been implicated in the pathogenesis and progression of preeclampsia (Zhang et al., 2020; Oancea et al., 2025). Among these, miR-7641 has been shown to regulate cell proliferation and apoptosis in several tumor models, with validated target genes in that context including RPS16, TNFSF10, and ARID1A (Jeong et al., 2017; Yang et al., 2020); however, its function in placental tissue has not been fully elucidated. Bioinformatic prediction has identified a putative binding site between miR-7641 and the 3′-UTR of GPX4, suggesting its potential involvement in the regulation of ferroptosis-related pathways (**Supplementary Figure 1**). Nevertheless, the expression profile of miR-7641 in SPE placentas, its relationship with ferroptosis-related molecular phenotypes, and its potential clinical utility in risk stratification remain unexplored.

Our group previously conducted a preliminary mechanistic investigation of miR-7641 in trophoblast ferroptosis (Hu et al., 2025). In that study, Western blot analysis confirmed upregulation of COX2 and downregulation of GPX4 at the protein level in SPE placental tissue. *In vitro* experiments using an H_2_O_2_-induced trophoblast oxidative stress model demonstrated that miR-7641 mimic transfection significantly altered trophoblast proliferation, apoptosis, invasion, and migration. Target gene prediction and dual-luciferase reporter assays provided preliminary evidence for the direct targeting activity of miR-7641. These findings established a mechanistic basis linking miR-7641 to trophoblast ferroptosis in preeclampsia. However, that study was limited by a small clinical sample size (n = 60), the absence of EOPE/LOPE subtype stratification, and the lack of assessment of whether miR-7641 and ferroptosis-related markers confer clinical risk stratification value.

The mechanistic evidence described above was established in our prior work (Hu et al., 2025). The present study addresses the unanswered clinical translational questions by employing a single-center retrospective clinicopathological design and a clinical epidemiological analytic framework. In a larger cohort of SPE patients and normotensive controls (n = 134), placental miR-7641, MDA, COX2, and GPX4 levels were simultaneously quantified at the cohort level alongside comprehensive clinical data. This study was designed to address three specific questions: (1) whether miR-7641 upregulation in SPE placentas is associated with ferroptosis-related phenotypic alterations, and whether this association persists within the SPE subgroup after removing between-group confounding effects; (2) whether EOPE and LOPE exhibit distinct molecular burden profiles consistent with their established clinical heterogeneity; and (3) whether the addition of placental molecular markers to MAP provides statistically significant incremental value for SPE risk stratification, as assessed by the net reclassification improvement (NRI) and integrated discrimination improvement (IDI)—metrics that quantify how much a new predictor improves classification of individuals into clinically meaningful risk categories, beyond what is captured by the area under the ROC curve alone. This study is based on placental tissue obtained at delivery and primarily reflects the relationship between placental molecular phenotypes and clinical outcomes rather than serving as a mid-trimester screening tool; its clinical applicability requires further validation through integration with circulating biomarkers and prospective multicenter studies.

## Materials and methods

### Study design, reporting standards, and ethical approval

This was a single-center retrospective clinicopathological study, positioned as a clinical epidemiological extension of our group’s previously published mechanistic research on miR-7641 and placental ferroptosis, aimed at evaluating the incremental risk stratification value of the previously identified molecular markers in a larger clinical cohort. The manuscript was prepared in accordance with the STROBE guidelines (von Elm et al., 2007). The study protocol was reviewed and approved by the Ethics Committee of Yongkang First People’s Hospital. Given the retrospective design and complete data anonymization, the requirement for written informed consent was waived. The study was conducted in compliance with the principles of the Declaration of Helsinki (World Medical Association, 2013).

### Study population, diagnostic criteria, and exclusion criteria

A total of 134 women who underwent cesarean delivery at our institution between January 2023 and October 2024 were consecutively enrolled, comprising 64 with SPE and 70 normotensive controls. SPE was diagnosed according to the Chinese Guidelines for the Diagnosis and Management of Hypertensive Disorders in Pregnancy (2020) (Hypertensive Disorders in Pregnancy Subgroup, Chinese Society of Obstetrics and Gynecology, Chinese Medical Association, 2020) and the American College of Obstetricians and Gynecologists (ACOG) Practice Bulletin No. 222 (American College of Obstetricians and Gynecologists, 2020): new-onset hypertension after 20 weeks of gestation (systolic blood pressure ≥160 mmHg and/or diastolic blood pressure ≥110 mmHg) accompanied by proteinuria (24-hour urine protein ≥2.0 g or random urine protein ≥”++”) or any evidence of target organ damage. Controls were contemporaneous normotensive pregnant women with no hypertensive disorders of pregnancy or other serious complications.

Exclusion criteria included pre-existing chronic hypertension, diabetes mellitus, chronic kidney disease, or autoimmune disorders; multiple gestation; fetal chromosomal abnormalities or major congenital malformations; placental abruption, placenta previa, or active infection; and incomplete clinical records or unavailable placental specimens. As a retrospective exploratory study, the sample size was determined by case availability during the study period, and no *a priori* sample size calculation was performed.

### Clinical data collection and subtype classification

Clinical data were systematically extracted from the hospital electronic medical records, including baseline characteristics (age, BMI, gravidity, and parity), admission blood pressure and MAP, laboratory results, maternal outcomes (length of hospitalization and postpartum complications), and perinatal outcomes (gestational age at delivery, birth weight, Apgar scores, NICU admission rate, and FGR incidence). The SPE group was subdivided by gestational age at delivery into EOPE (<34 weeks, n = 21) and LOPE (≥34 weeks, n = 43). Given the limited sample size of the EOPE subgroup, all related analyses were designated as exploratory.

### Placental tissue specimen collection and processing

Placental specimens were collected immediately after cesarean delivery. Villous tissue blocks (approximately 1.0 cm³) were excised from the central region of the maternal surface, avoiding areas of infarction, organized thrombi, and calcification. After removal of blood clots and decidual remnants, specimens were rinsed with normal saline, blotted dry with sterile filter paper, snap-frozen in liquid nitrogen, and stored at −80 °C. All procedures were performed by trained personnel following a standardized protocol, with processing completed within 15 minutes of placental delivery.

### Quantification of placental miR-7641 by qRT-PCR

Total RNA was extracted from placental tissue using TRIzol™ Reagent (Cat. No. 15596018, Invitrogen, Thermo Fisher Scientific, Carlsbad, CA, USA), and concentration and purity were assessed by microspectrophotometry on a NanoDrop™ 2000 (Thermo Fisher Scientific, Waltham, MA, USA; A260/A280: 1.8–2.0). Following cDNA synthesis with the Mir-X™ miRNA First-Strand Synthesis Kit (Cat. No. 638313, Takara Bio Inc., Shiga, Japan) according to the manufacturer’s instructions, miR-7641 expression was quantified by qRT-PCR on an Applied Biosystems™ 7500 Fast Real-Time PCR System (Applied Biosystems, Foster City, CA, USA) using the Mir-X™ miRNA qRT-PCR TB Green^®^ Kit (Cat. No. 638314, Takara Bio Inc.), with U6 small nuclear RNA as the internal reference. Primer sequences (5′→3′) were: miR-7641 forward, TTGATCTCGGAAGCTAAGC; U6 forward, CTCGCTTCGGCAGCACA; U6 reverse, AACGCTTCACGAATTTGCGT; the mRQ 3′universal reverse primer supplied with the Mir-X™ kit was used for miR-7641. All primers were synthesized by Sangon Biotech Co., Ltd. (Shanghai, China). All reactions included negative controls, and each sample was assayed in at least technical duplicate. Mean Ct values were used, with a coefficient of variation <5% for technical replicates. Relative expression was calculated using the 2-ΔΔCt method (Livak and Schmittgen, 2001).

### Cohort-level quantification of ferroptosis-related markers

Approximately 50 mg of frozen placental tissue was homogenized in pre-cooled PBS (Cat. No. ST476, Beyotime Biotechnology, Shanghai, China) containing protease and phosphatase inhibitor cocktail (Cat. No. P1050, Beyotime Biotechnology), and total protein concentration was determined by the Enhanced BCA Protein Assay (Cat. No. P0010, Beyotime Biotechnology) for normalization. COX2 was measured using a Human PTGS2/COX-2 ELISA Kit (Cat. No. E-EL-H1846, Elabscience Biotechnology Co., Ltd., Wuhan, China) and GPX4 using a Human GPX4 ELISA Kit (Cat. No. EH8916, Wuhan Fine Biotech Co., Ltd., Wuhan, China); both were normalized to ng/mg protein. MDA was quantified using a TBA colorimetric assay kit (Cat. No. A003-1-2, Nanjing Jiancheng Bioengineering Institute, Nanjing, China), expressed as nmol/mg protein.

ELISA rather than Western blot was selected for cohort-level quantification for the following reasons: ELISA provides standardized continuous data suitable for regression modeling and NRI/IDI incremental assessment across 134 specimens (Shi et al., 2024), whereas Western blot is a semi-quantitative method unsuitable for large-sample statistical modeling. Furthermore, COX2 upregulation and GPX4 downregulation in SPE placentas were previously verified by Western blot in our mechanistic study; the present study was designed to quantify incremental risk stratification value rather than to replicate protein expression validation. The above markers serve as cohort-level quantitative surrogate indicators of ferroptosis-related biochemical phenotypes and do not constitute direct evidence of ferroptosis.

### Statistical analysis

Statistical analyses were performed using SPSS 27.0 and R 4.2.1. Normally distributed continuous variables are presented as mean ± SD (Student’s t-test), non-normally distributed data as median (interquartile range) (Mann–Whitney U test), and categorical variables as frequency (%) (chi-square test or Fisher’s exact test). Between-group comparisons report mean differences with 95% confidence intervals. All tests were two-sided, with P < 0.05 considered statistically significant. Spearman correlation analyses report correlation coefficients with 95% confidence intervals and were performed in both the overall cohort and the SPE subgroup; the latter was intended to exclude spurious correlations driven by between-group mean differences. Multivariable logistic regression was constructed with SPE occurrence as the dependent variable. Variables with univariable P < 0.10 and clinical plausibility were included, with variance inflation factor (VIF) < 5 as the preset threshold for exclusion due to multicollinearity. Model fit was evaluated by the Hosmer–Lemeshow test. Statistical analyses were structured across three tiers: primary analyses comprised between-group comparisons and multivariable regression; secondary analyses comprised incremental value testing and overall-cohort correlations; and exploratory analyses comprised EOPE/LOPE subgroup comparisons and within-SPE correlations.

To evaluate the incremental value of molecular markers beyond MAP, nested models were constructed: Model 1 included MAP alone, and Model 2 added the retained molecular markers. Incremental discrimination was assessed using likelihood ratio tests, NRI, IDI (nricens package; 1,000 bootstrap iterations), and the DeLong test (DeLong et al., 1988). Because the continuous NRI may overestimate reclassification improvement, event and non-event NRI components are reported separately. These models were designed to assess incremental associative value and, having not undergone external validation, should not be interpreted as clinical prediction tools. Prespecified sensitivity analyses included: adjustment for gestational age to evaluate confounding; quartile analysis of miR-7641 to assess dose–response relationships; and repetition of the primary analysis after excluding cases with severe placental complications.

## Results

### Participant selection and baseline characteristics

**Figure 1:**
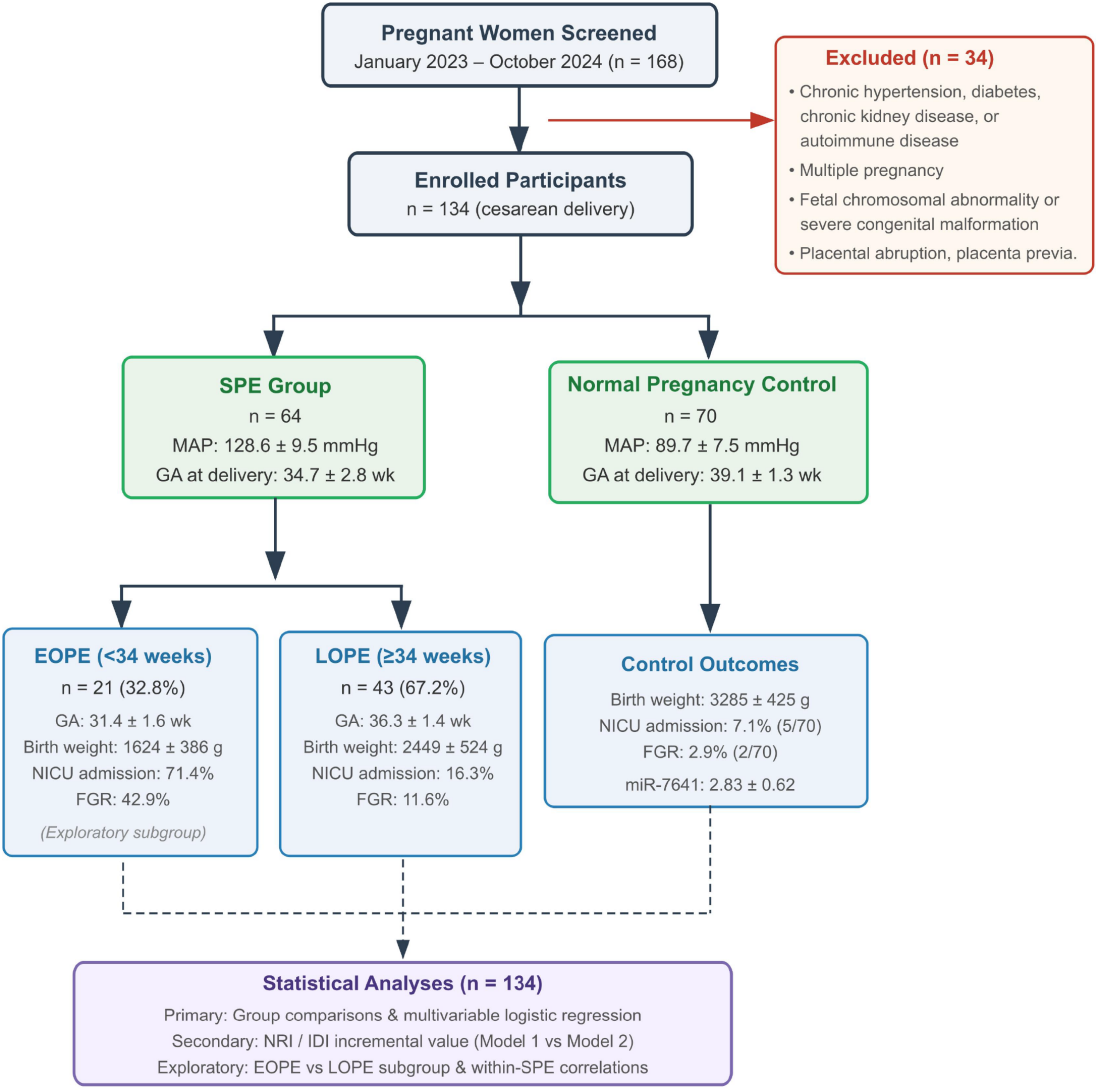
Study participant flowchart. Of 168 screened women, 134 were enrolled and classified into SPE (n = 64) and Control (n = 70), with SPE subdivided into EOPE (n = 21) and LOPE (n = 43). Exclusion criteria are shown; full criteria are detailed in Section 2.2.

Of 168 women screened during the study period, 34 were excluded, yielding a final cohort of 134 (SPE, n = 64; controls, n = 70), including 21 EOPE (32.8%) and 43 LOPE (67.2%). The two groups were comparable in age, pre-pregnancy BMI, gravidity, and parity (all P > 0.05). MAP was significantly higher in the SPE group than in controls (128.6 ± 9.5 vs. 89.7 ± 7.5 mmHg; mean difference: 38.9 mmHg, 95% CI: 35.2–42.6, P < 0.001), with earlier gestational age at delivery, lower birth weight, and significantly higher rates of NICU admission (34.4% vs. 7.1%) and FGR (21.9% vs. 2.9%) (**Table 1**, **Figure 1**).

**Table 1:** Baseline characteristics, placental molecular markers, and maternal-fetal outcomes.

Variable	Control (n = 70)	SPE (n = 64)	P	EOPE (n = 21)	LOPE (n = 43)	P
Demographics						
Age, years	29.4 ± 4.2	30.1 ± 4.5	0.358	30.6 ± 4.8	29.8 ± 4.4	0.512
Pre-pregnancy BMI, kg/m^2^	23.1 ± 3.4	23.8 ± 3.6	0.246	24.2 ± 3.9	23.6 ± 3.5	0.538
Gravidity, median (IQR)	2 (1–3)	2 (1–3)	0.712	2 (1–3)	2 (1–3)	0.846
Nulliparity, n (%)	45 (64.3)	38 (59.4)	0.563	13 (61.9)	25 (58.1)	0.774
Clinical parameters						
MAP, mmHg	89.7 ± 7.5	128.6 ± 9.5	<0.001	131.4 ± 10.2	127.2 ± 9.0	0.103
GA at delivery, weeks	39.1 ± 1.3	34.7 ± 2.8	<0.001	31.4 ± 1.6	36.3 ± 1.4	<0.001
Birth weight, g	3285 ± 425	2178 ± 682	<0.001	1624 ± 386	2449 ± 524	<0.001
NICU admission, n (%)	5 (7.1)	22 (34.4)	<0.001	15 (71.4)	7 (16.3)	<0.001
FGR, n (%)	2 (2.9)	14 (21.9)	<0.001	9 (42.9)	5 (11.6)	0.005
Proteinuria ≥2+ or ≥2.0 g, n (%)	—	51 (79.7)	—	18 (85.7)	33 (76.7)	0.532
Placental molecular markers						
miR-7641 (relative expression)	2.83 ± 0.62	5.81 ± 0.89	<0.001	6.45 ± 0.88	5.49 ± 0.75	<0.001
MDA, nmol/mg protein	3.24 ± 1.15	6.47 ± 1.82	<0.001	7.38 ± 1.98	6.02 ± 1.62	0.006
COX2, ng/mg protein	8.34 ± 2.71	14.58 ± 4.12	<0.001	16.52 ± 4.25	13.63 ± 3.82	0.012
GPX4, ng/mg protein	22.51 ± 5.34	13.78 ± 4.56	<0.001	10.82 ± 3.92	15.23 ± 4.38	<0.001

SPE, severe preeclampsia; EOPE, early-onset PE (<34 weeks); LOPE, late-onset PE (≥34 weeks); BMI, body mass index; MAP, mean arterial pressure; GA, gestational age; NICU, neonatal intensive care unit; FGR, fetal growth restriction; MDA, malondialdehyde; COX2, cyclooxygenase-2; GPX4, glutathione peroxidase 4. Continuous variables: mean ± SD (*t*-test) or median (IQR) (Mann–Whitney *U*); categorical variables: *n* (%) (χ^2^/Fisher exact). EOPE vs LOPE Cohen’s *d*: miR-7641 = 1.18, MDA = 0.81, GPX4 = 1.07; EOPE subgroup (*n* = 21) comparisons are exploratory.

### Placental miR-7641 and ferroptosis-related markers: SPE versus controls

**Figure 2:**
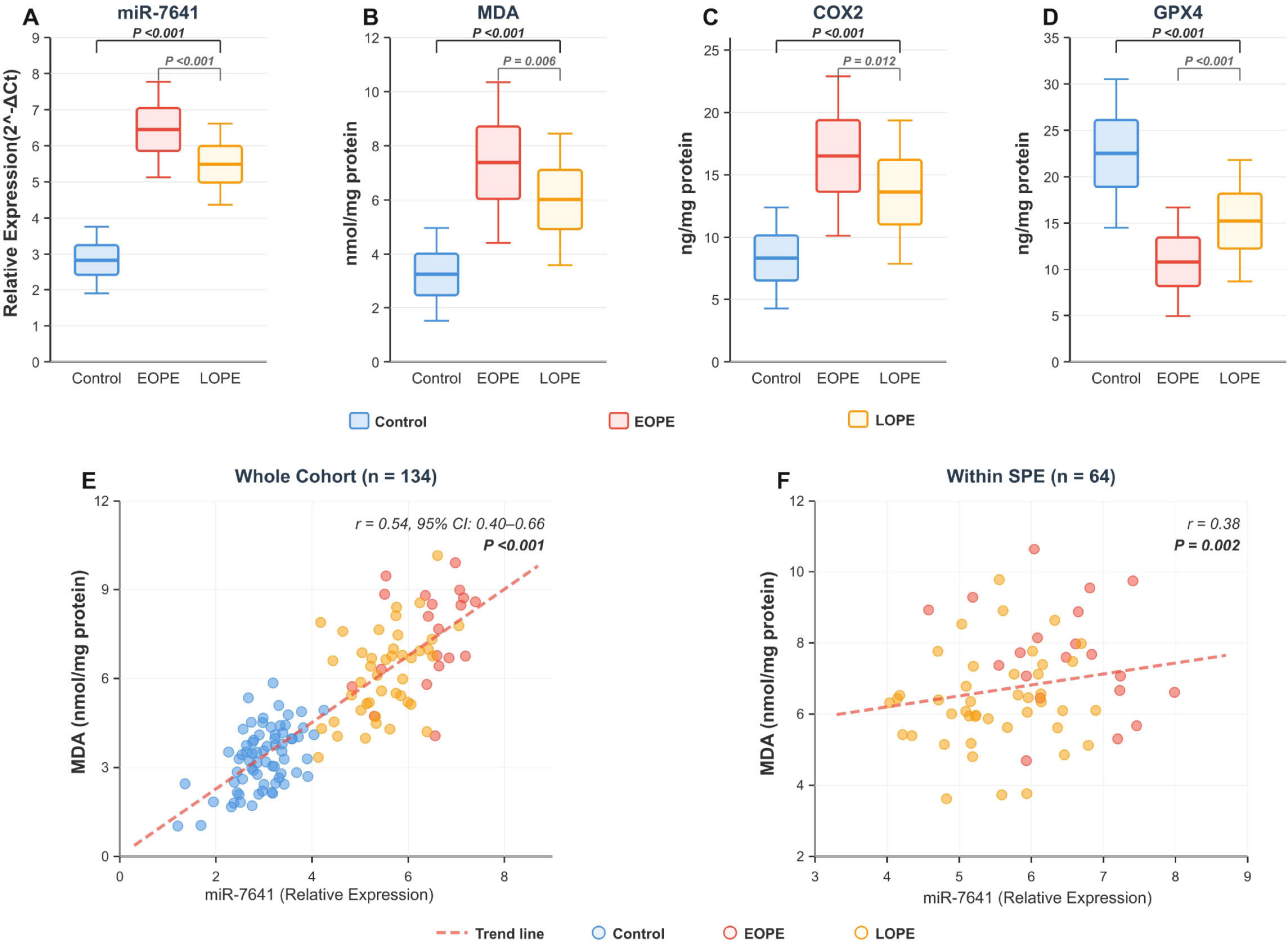
Placental molecular markers and miR-7641–MDA correlation. miR-7641 was quantified by qRT-PCR (2-ΔΔCt, normalized to U6); MDA, COX2, and GPX4 by ELISA. Group comparisons used two-sample t-tests (Control vs SPE; EOPE vs LOPE). Correlations were assessed using Spearman’s rank correlation coefficient, with dashed lines indicating linear trend fits. In **(A–D)**, upper brackets denote Control vs entire SPE group; lower brackets denote EOPE vs LOPE. **(E)** shows the correlation between placental miR-7641 relative expression and MDA levels in the whole cohort (n = 134). **(F)** includes EOPE and LOPE only.

Compared with controls, SPE placentas exhibited significantly elevated miR-7641 levels (5.81 ± 0.89 vs. 2.83 ± 0.62; mean difference: 2.98, 95% CI: 2.72–3.24, P < 0.001). MDA and COX2 were also significantly elevated, and GPX4 was significantly reduced (all P < 0.001), revealing a ferroptosis-related molecular phenotype characterized by miR-7641 upregulation, exacerbated lipid peroxidation, and GPX4 downregulation. Specific values and effect sizes for each marker are detailed in **Table 1** (**Figures 2A–D**).

### Molecular phenotype and clinical differences between EOPE and LOPE

Compared with LOPE, the EOPE subgroup exhibited higher miR-7641 levels (6.45 ± 0.88 vs. 5.49 ± 0.75; Cohen’s d = 1.18), higher MDA (Cohen’s d = 0.81), and lower GPX4 (Cohen’s d = 1.07), with all three effect sizes exceeding 0.8, indicating clinically meaningful between-group differences. COX2 was also significantly elevated (P = 0.012), although its effect size was relatively smaller than those of the other three markers. The EOPE subgroup also had higher rates of FGR (42.9% vs. 11.6%) and NICU admission (71.4% vs. 16.3%). Given that the EOPE subgroup comprised only 21 cases, these results should be considered exploratory (**Table 1**).

### Correlations of miR-7641 with ferroptosis markers and maternal–fetal outcomes

In the overall cohort, miR-7641 was positively correlated with MDA (r = 0.54, 95% CI: 0.40–0.66) and COX2 (r = 0.51), and negatively correlated with GPX4 (r = −0.49), gestational age at delivery (r = −0.46), and birth weight (r = −0.42) (all P < 0.001). In the within-SPE validation, the associations of miR-7641 with MDA (r = 0.38, P = 0.002) and GPX4 (r = −0.34, P = 0.006) persisted but were attenuated, indicating that the overall-cohort correlations were partly driven by between-group mean differences while an independent within-group association remained. The correlation between miR-7641 and COX2 did not reach statistical significance within the SPE group (r = 0.24, P = 0.054) (**Figures 2E, F**).

### Multivariable logistic regression

**Figure 3:**
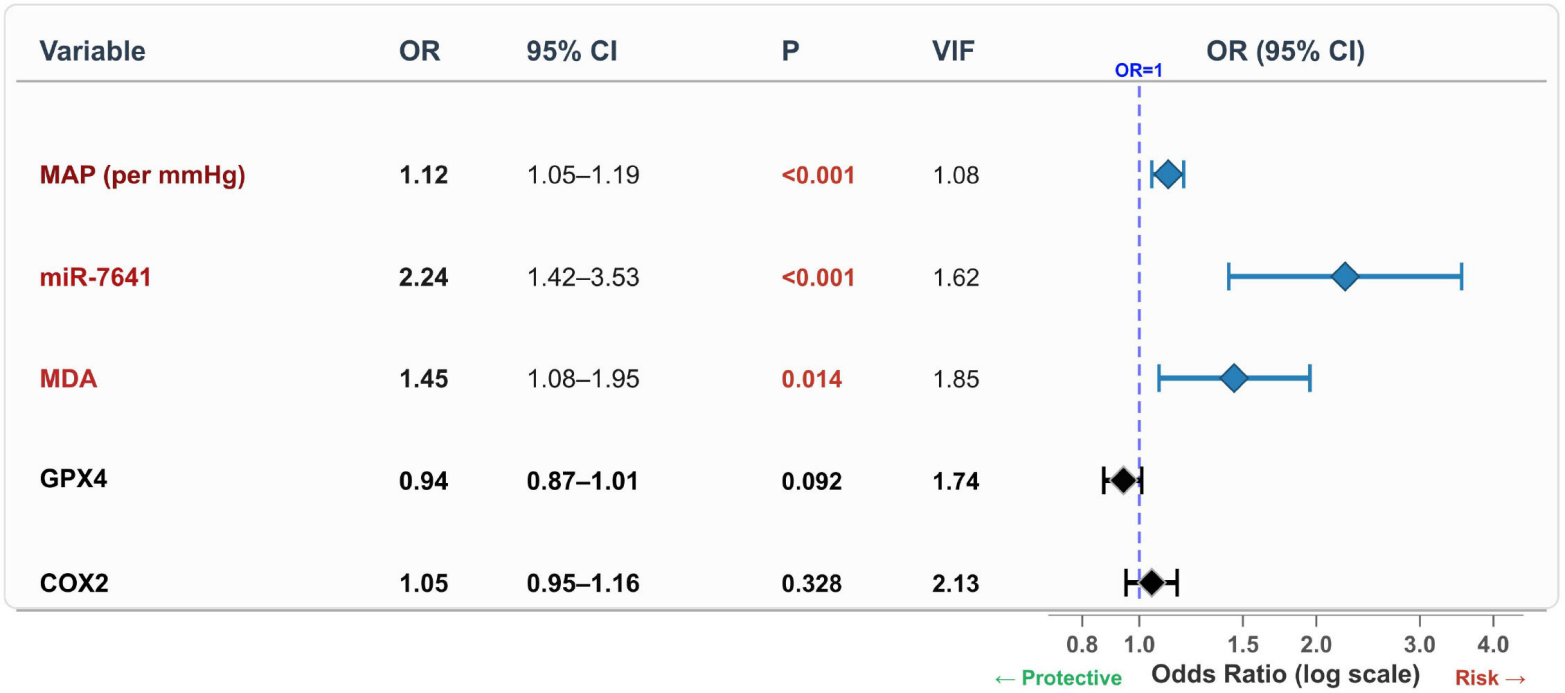
Forest plot of multivariable logistic regression for SPE (n = 134). MAP, miR-7641, and MDA were independently associated with SPE after adjustment for all five candidates. Odds ratios with 95% CIs are shown on a logarithmic scale; significant associations (P < 0.05) are highlighted. Hosmer–Lemeshow χ² = 6.14, P = 0.624; all VIF < 2.5.

In multivariable analysis, MAP (OR = 1.12, 95% CI: 1.05–1.19), miR-7641 (OR = 2.24, 95% CI: 1.42–3.53), and MDA (OR = 1.45, 95% CI: 1.08–1.95) were independently associated with SPE (all P < 0.05). GPX4 exhibited a protective trend that did not reach significance (OR = 0.94, P = 0.092), and COX2 showed no independent association after adjustment. All VIF values were <2.5; the Hosmer–Lemeshow test yielded P = 0.624 (**Table 2**, **Figure 3**).

**Table 2:** Multivariable logistic regression for SPE (upper) and nested model incremental value assessment (lower).

Panel A: multivariable logistic regression					
Variable	Univariable OR (95% CI)	P	Multivariable OR (95% CI)	P	VIF
MAP, per mmHg	1.15 (1.09–1.22)	<0.001	1.12 (1.05–1.19)	<0.001	1.08
miR-7641	3.86 (2.54–5.87)	<0.001	2.24 (1.42–3.53)	<0.001	1.62
MDA	1.82 (1.44–2.30)	<0.001	1.45 (1.08–1.95)	0.014	1.85
GPX4	0.85 (0.79–0.91)	<0.001	0.94 (0.87–1.01)	0.092	1.74
COX2	1.18 (1.08–1.29)	<0.001	1.05 (0.95–1.16)	0.328	2.13

Panel B: nested model incremental discrimination				
Metric	Model 1 (MAP alone)	Model 2(MAP + miR-7641 + MDA)	Difference (95% CI)	P
AUC (95% CI)	0.843 (0.774–0.912)	0.906 (0.854–0.958)	ΔAUC = 0.063	0.008
Continuous NRI	—	—	0.573 (0.314–0.826)	<0.001
Event NRI	—	—	0.344	—
Non-event NRI	—	—	0.229	—
IDI (95% CI)	—	—	0.112 (0.064–0.168)	<0.001

Hosmer–Lemeshow goodness-of-fit: χ2 = 6.14, P = 0.624.

OR, odds ratio; CI, confidence interval; VIF, variance inflation factor; AUC, area under the ROC curve; NRI, net reclassification improvement; IDI, integrated discrimination improvement. Univariable screening threshold P < 0.10; multivariable model adjusted for all five predictors (all VIF < 2.5). Model 2 retains independently significant predictors only. Continuous NRI via 1000-fold bootstrap; event and non-event components reported to address overestimation bias. Model AUC at n = 134 may carry optimistic bias; values reflect relative incremental improvement and require external validation.

### Incremental risk stratification value of molecular markers beyond MAP

Model 2 (MAP + miR-7641 + MDA) improved the AUC from 0.843 to 0.906 compared with Model 1 (MAP alone) (ΔAUC = 0.063, DeLong P = 0.008), with the likelihood ratio test yielding P < 0.001. The continuous NRI was 0.573 (95% CI: 0.314–0.826, P < 0.001), with an event NRI component of 0.344 and a non-event NRI component of 0.229, indicating improved risk classification at both ends. The IDI was 0.112 (95% CI: 0.064–0.168, P < 0.001). It should be noted that the model AUC at n = 134 may be subject to optimistic bias, and the primary value of NRI/IDI lies in the relative improvement between models rather than in absolute predictive performance (**Table 2**).

### Sensitivity analyses

After inclusion of gestational age, the OR for miR-7641 decreased from 2.24 to 1.86 (P = 0.016), and the OR for MDA decreased from 1.45 to 1.32 (P = 0.042), suggesting that gestational age partially mediates but does not fully account for the association between molecular markers and SPE. Quartile analysis of miR-7641 revealed a progressive increase in ORs from Q1 to Q4 (P for trend < 0.001), supporting a dose–response relationship. After exclusion of six cases with severe placental complications, the primary conclusions remained robust (NRI = 0.548, IDI = 0.104, both P < 0.001).

## Discussion

### Principal findings

In a cohort of 134 women, this study systematically examined the associations of placental miR-7641 and ferroptosis-related markers with SPE clinical subtypes and maternal–fetal outcomes, and rigorously evaluated their incremental risk stratification value beyond MAP. The principal findings are as follows: SPE placentas exhibited a ferroptosis-related molecular phenotype characterized by miR-7641 upregulation, elevated MDA and COX2, and reduced GPX4, with more pronounced alterations in the EOPE subgroup. The associations between miR-7641 and ferroptosis markers persisted in the within-SPE validation, excluding the possibility of spurious correlations driven by between-group mean differences (Kievit et al., 2013). The addition of miR-7641 and MDA to the MAP baseline model yielded statistically significant NRI and IDI, demonstrating that placental molecular markers provide incremental information beyond conventional blood pressure parameters.

### Consistency of ferroptosis-related molecular phenotype with prior mechanistic evidence

The core pathology of preeclampsia involves placental hypoperfusion, ischemia–reperfusion injury, and oxidative stress (Guerby et al., 2021). Systematic reviews have demonstrated that lipid peroxidation products are significantly elevated in the placentas and blood of preeclamptic women, with reduced activity of antioxidant enzymes such as GPx and MDA levels correlating with disease severity (Freire et al., 2023). Ferroptosis, marked by lipid peroxidation accumulation and GPX4 dysfunction, has been implicated in impaired spiral artery remodeling in preeclampsia through disrupted iron metabolic homeostasis (Yang et al., 2022). Recent studies have further revealed that Nox2 can regulate trophoblast ferroptosis via the STAT3/GPX4 pathway, with targeted Nox2 inhibition representing a potential therapeutic strategy for preeclampsia (Xu et al., 2024). The pattern of miR-7641 upregulation, MDA accumulation, and GPX4 downregulation observed in the present study is directionally consistent with the accumulating evidence for a role of ferroptosis in placental injury in preeclampsia, and aligns with prior reports by Park et al. and Zhang et al. on ferroptosis features in preeclamptic placentas and miRNA-mediated trophoblast ferroptosis, respectively.

Our group’s prior mechanistic study confirmed COX2 upregulation and GPX4 downregulation in SPE placentas by Western blot, validated the effects of miR-7641 on trophoblast proliferation, apoptosis, and invasion in an H_2_O_2_-induced model, and provided preliminary evidence of direct targeting through dual-luciferase reporter assays. In the present larger clinical cohort, the negative correlation between miR-7641 and GPX4 persisted within the SPE group (r = −0.34, 95% CI: −0.55 to −0.10, P = 0.006), directionally consistent with the mechanistic findings. The two studies were deliberately designed with distinct roles—the prior study provided protein-level and cellular functional mechanistic verification, while the present study evaluated incremental risk stratification value in a larger cohort—representing an intentional progression from mechanistic discovery to clinical translation, with the two forming a complementary chain of evidence.

It must be emphasized that MDA, COX2, and GPX4 in the present study serve as surrogate indicators of ferroptosis-related phenotypes and cannot confirm the actual occurrence of ferroptosis (Wang et al., 2019). The negative correlation between miR-7641 and GPX4 is an observational association and does not equate to a direct regulatory relationship. Multiple recent reviews have stressed that GPX4 downregulation and MDA elevation alone are insufficient to definitively establish ferroptosis, which requires convergent evidence from ultrastructural analysis, iron metabolism profiling, and genetic perturbation studies (Ng et al., 2025). Accordingly, the term “ferroptosis-related molecular phenotype” rather than “ferroptosis” is used throughout to maintain alignment between the terminology and the level of evidence.

### Differential molecular burden between EOPE and LOPE and its clinical significance

EOPE typically presents with more pronounced placental-origin features, including prominent perfusion defects and dysfunction, whereas LOPE is more frequently associated with maternal metabolic derangements and pre-existing cardiovascular risk factors (Hu et al., 2022). Transcriptomic studies have provided molecular-level confirmation that the two subtypes involve distinct placental pathological mechanisms: EOPE is predominantly characterized by metabolic and transport pathway abnormalities, while LOPE features immune-related pathway dysregulation (Ren et al., 2021). Single-cell and spatial multi-omics analyses have further revealed upregulated angiogenic signaling pathways and alterations in Hofbauer cell subpopulations in EOPE, whereas LOPE maintains a relatively intact cellular composition but exhibits a stronger inflammatory transcriptional program (Admati et al., 2023). In the present study, the EOPE subgroup exhibited higher miR-7641 (Cohen’s d = 1.18), greater MDA accumulation (Cohen’s d = 0.81), and lower GPX4 levels (Cohen’s d = 1.07), accompanied by higher rates of FGR and NICU admission, displaying a consistent pattern wherein greater molecular burden corresponds to poorer perinatal outcomes. All effect sizes qualified as large (Cohen’s d > 0.8) (Cohen, 1988), suggesting that the between-group differences are not only statistically significant but also of clinically meaningful magnitude. This profile is consistent with the pathological basis of EOPE as a placental-origin disease and suggests that incorporating placental molecular information into the SPE stratification framework may facilitate a transition from purely clinical phenotyping to integrated clinical–molecular stratification.

The EOPE subgroup comprised only 21 cases, limiting statistical power, and these findings are therefore designated as exploratory. The extent to which molecular marker differences are independent of gestational age per se requires further clarification through interaction analyses in larger samples.

### Clinical interpretation of incremental value

Given that all molecular markers were measured in placental tissue obtained at delivery, these findings should be interpreted as providing proof of concept for biological and clinicopathological stratification rather than as evidence of a clinically deployable mid-pregnancy screening tool. The addition of miR-7641 and MDA to the MAP baseline model improved the AUC from 0.843 to 0.906 (DeLong P = 0.008), with an NRI of 0.573 and an IDI of 0.112, all statistically significant, indicating that placental molecular markers carry complementary risk information not captured by MAP alone. Decomposition of the NRI revealed that, following addition of the molecular markers, 34.4% of SPE patients were correctly reclassified into a higher risk category (event NRI component: 0.344), and 22.9% of controls were correctly reclassified into a lower category (non-event NRI component: 0.229), demonstrating improved risk classification at both ends.

These results warrant cautious interpretation. The model AUC at n = 134 may be subject to optimistic bias. The continuous NRI has a methodological tendency to overestimate reclassification improvement (Leening et al., 2014) and may even yield false-positive results for uninformative markers (Pepe et al., 2015); accordingly, event and non-event NRI components are reported separately to enhance interpretability. After adjustment for gestational age, the effects were attenuated but remained independent, suggesting that the incremental value is not entirely driven by gestational age confounding, although partial mediation implies that the true increment may be slightly smaller than estimated herein. Most importantly, all markers were derived from placental tissue obtained at delivery and cannot be obtained non-invasively during pregnancy. The current model is positioned as proof of concept and variable selection—demonstrating that these molecular markers carry valuable risk information and providing candidate variables and modeling directions for future exploration of accessible markers such as circulating miRNAs (Ghosh et al., 2024)—rather than as a prediction tool ready for clinical decision-making.

Clinical translation of these findings will require prospective validation using accessible circulating biomarkers. Priority candidates include blood-based miRNAs—in particular extracellular vesicle–encapsulated miR-7641—and oxidative stress–related indicators such as plasma MDA, benchmarked against established antenatal predictors such as the sFlt-1/PlGF ratio. Recent work demonstrating that maternal circulating extracellular vesicle–derived miRNA panels can achieve early prediction prior to the onset of preeclampsia symptoms (Ghosh et al., 2024) supports the feasibility of this pathway. Accordingly, the present placental findings should be regarded primarily as identification of biologically relevant candidate markers for subsequent non-invasive validation in multicenter prospective studies.

### Strengths and limitations

This study simultaneously obtained four placental molecular markers and comprehensive clinical data within a single cohort, validated associations through within-SPE correlations to guard against Simpson’s paradox (Kievit et al., 2013), and quantified the incremental value of molecular markers beyond MAP using the NRI/IDI framework. The previously published mechanistic study provides independent experimental support for the clinical observations reported herein.

This was a single-center retrospective study with a limited sample size, and the EOPE subgroup comprised only 21 cases; all EOPE/LOPE comparisons are therefore explicitly designated as exploratory and hypothesis-generating, and replication in larger cohorts will be required before subtype-specific conclusions can be drawn. All markers were derived from post-delivery placental tissue and cannot be obtained non-invasively during pregnancy; thus, the model is positioned as proof of concept. Ferroptosis-related phenotype characterization relied on cohort-level quantitative data without ultrastructural or iron metabolism markers (Ng et al., 2025); accordingly, the term “ferroptosis-related molecular phenotype” is used throughout to match the level of evidence. Protein-level validation and target gene functional verification were completed in the prior mechanistic study; the present study was intentionally positioned as its clinical epidemiological extension, with the two studies being methodologically complementary rather than redundant. Validation of these findings in larger, prospective, multicenter cohorts will be essential, together with evaluation of non-invasive circulating miR-7641 and related biomarkers (Ghosh et al., 2024).

## Conclusions

SPE placentas exhibit a ferroptosis-related molecular phenotype centered on miR-7641 upregulation and GPX4 downregulation, which is more pronounced in EOPE. The addition of miR-7641 and MDA to MAP yielded statistically significant NRI and IDI, demonstrating that placental molecular markers provide incremental risk stratification information beyond conventional blood pressure parameters at the delivery time point. These findings constitute proof of concept and should not be interpreted as evidence of a mid-pregnancy screening tool; clinical translation awaits prospective validation using accessible circulating biomarkers in multicenter cohorts.

## Data Availability

The raw data supporting the conclusions of this article will be made available by the authors, without undue reservation.
