# Supplementary material for: Incremental value of placental miR-7641 and ferroptosis-related phenotypes in risk stratification of severe preeclampsia
Source: Front Physiol. 2026 May 13;17:1834708. doi: 10.3389/fphys.2026.1834708 (PMC13212197; doi:10.3389/fphys.2026.1834708)
Supplement: Supplementary file 1 [file SupplementaryFile1.docx]

**Supplementary Figure S1**


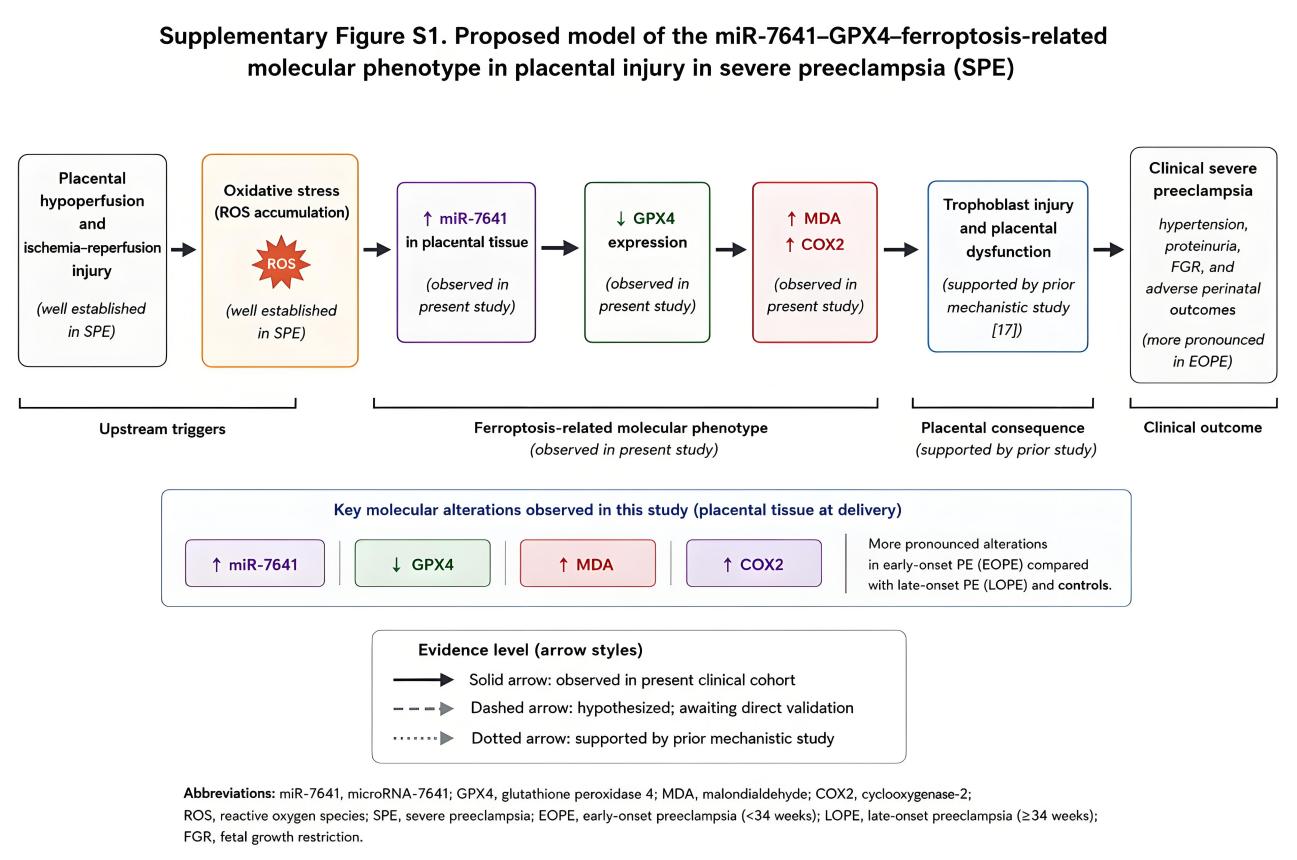


**Supplementary Figure S1. Proposed model of the miR-7641–GPX4 axis and ferroptosis-related molecular phenotype in placental injury in severe preeclampsia (SPE).** Placental hypoperfusion and oxidative stress drive miR-7641 upregulation, which is hypothesized to suppress GPX4 via 3′-UTR binding, leading to MDA accumulation and COX2 upregulation. This phenotype is proposed to contribute to trophoblast injury and clinical SPE, with more pronounced alterations in early-onset disease. The schematic summarizes the proposed sequence and does not itself constitute mechanistic evidence.
